# Core Proteomics and Immunoinformatic Approaches to Design a Multiepitope Reverse Vaccine Candidate against Chagas Disease

**DOI:** 10.3390/vaccines10101669

**Published:** 2022-10-07

**Authors:** Sk Injamamul Islam, Saloa Sanjida, Sheikh Sunzid Ahmed, Mazen Almehmadi, Mamdouh Allahyani, Abdulelah Aljuaid, Ahad Amer Alsaiari, Mustafa Halawi

**Affiliations:** 1 The International Graduate Program of Veterinary Science and Technology (VST), Department of Veterinary Microbiology, Faculty of Veterinary Science and Technology, Chulalongkorn University, Bangkok 10330, Thailand; 2 Department of Environmental Science and Technology, Faculty of Applied Science and Technology, Jashore University of Science and Technology, Jashore 7408, Bangladesh; 3 Department of Botany, Faculty of Biological Sciences, University of Dhaka, Dhaka 1000, Bangladesh; 4 Department of Clinical Laboratory Sciences, College of Applied Medical Sciences, Taif University, Taif 21944, Saudi Arabia; 5 Department of Medical Laboratory Technology, College of Applied Medical Sciences, Jazan University, Jazan 54943, Saudi Arabia

**Keywords:** *Trypanosoma cruzi*, proteomics, vaccine, epitopes, MD simulation

## Abstract

Chagas disease is a tropical ailment indigenous to South America and caused by the protozoan parasite *Trypanosoma cruzi*, which has serious health consequences globally. Insect vectors transmit the parasite and, due to the lack of vaccine availability and limited treatment options, we implemented an integrated core proteomics analysis to design a reverse vaccine candidate based on immune epitopes for disease control. Firstly, *T. cruzi* core proteomics was used to identify immunodominant epitopes. Therefore, we designed the vaccine sequence to be non-allergic, antigenic, immunogenic, and to have better solubility. After predicting the tertiary structure, docking and molecular dynamics simulation (MDS) were performed with TLR4, MHC-I, and MHC-II receptors to discover the binding affinities. The final vaccine design demonstrated significant hydrogen bond interactions upon docking with TLR4, MHC-I, and MHC-II receptors. This indicated the efficacy of the vaccine candidate. A server-based immune simulation approach was generated to predict the efficacy. Significant structural compactness and binding stability were found based on MDS. Finally, by optimizing codons on *Escherichia coli* K12, a high GC content and CAI value were obtained, which were then incorporated into the cloning vector pET2+ (a). Thus, the developed vaccine sequence may be a viable therapy option for Chagas disease.

## 1. Introduction

*Trypanosoma cruzi* is responsible for causing Chagas disease, which is spread by hematophagous insects and results in potentially fatal cardiac and gastrointestinal illnesses in humans [1]. *T. cruzi* transmission and morbidity have historically been centered in rural Latin American locations where poor housing conditions facilitate vectors of infestation. It is a parasitic disease that affects roughly 20 million people, mainly in Central and South America’s tropical areas. Furthermore, most *T. cruzi*-infected people in Central America are immigrants from Latin American endemic areas [1]. About 6–7 million people worldwide, mostly in Latin America, are estimated to be infected with *T. cruzi*, the parasite that causes Chagas disease [2]. Infected persons have been transported to cities inside and outside Latin America, including Canada and Europe, in recent decades [3,4,5]. WHO recognized Chagas disease as a neglected tropical disease (NTD) in 2005. This facilitated a greater recognition of the disease as a public health problem on the international scene and facilitated the fight against misinformation, the lack of social demand, and the weak political commitment to solve the problems related to Chagas disease.

It is a kinetoplastid protozoan that infects vertebrates and invertebrates at certain times throughout its life cycle [4]. When a triatomine vector acquires a blood meal from a mammalian host infected with the parasite, it consumes the circulating trypomastigotes [3]. Trypomastigotes, which are found in the vector’s midgut, give rise to epimastigotes, which are the most important reproducing stage in the invertebrate hosts [6]. Epimastigotes move to the hindgut, where they develop into infective metacyclic trypomastigotes, which are expelled with the vector’s feces. Metacyclic trypomastigotes enter the mammalian host by a bite wound or intact mucous membrane and infiltrate various nucleated cells via a lysosome-mediated process [7]. Trypomastigotes in the cytoplasm develop into intracellular amastigotes, which reproduce for 4 to 5 days with a doubling time of roughly 12 h. Once this period has passed, the amastigotes undergo a transformation into trypomastigotes, the host cell ruptures, and the trypomastigotes are discharged into circulation. At this point, the migratory pathogens can infect prey vectors, enter cells, and begin new replication cycles. Without effective antitrypanosomal therapy, the mammalian host is infected for the rest of its life [1].

Chagas disease is spread through the bite of the triatomine bug, a parasitic insect. It is prevalent in Latin American rural communities. Chagas disease consequences are a leading cause of cardiac mortality and disability in that part of the world, as well as impairment from both heart and gastrointestinal illness [8]. In the first stage of the disease, high levels of parasitemia and atypical symptoms of infection are present. After this acute stage, patients enter a chronic phase where they may have no symptoms, while parasitemia appears to be under control [9]. However, 20–40% of people will develop clinical symptoms many years after the initial infection, the most frequent of which is chronic Chagasic cardiomyopathy (CCC). Arrhythmias of increasing severity define CCC, leading to heart failure and death [10].

Tc52, one of the proteins secreted by *T. cruzi*, is a virulence factor that plays a vital role in the infection process. Scientists used a combination of in vitro and in vivo methodologies to demonstrate its function in the progression of infection and performed molecular profiling of the Tc52 minimum sequence with an immunosuppressive effect using a peptide sequence. The discovery of this factor’s biological action improves the chances of creating vaccines or medications to fight against *T. cruzi* [10,11]. *T. cruzi*, which causes Chagas disease, has five members of the Ribosomal P protein family [12]. These proteins are a group of proteins that create a projecting area in the ribosome’s large subunit. These proteins are thought to operate as a molecular switch in the ribosome, executing ratchet-like actions linked to elongation factor binding and release, and GTP hydrolysis [12]. Additionally, this protein has a role in the pathogenesis of Chagas disease, which is caused by *T. cruzi* [13].

While a therapeutic vaccine would be an attractive alternative, more strategies and drugs are still needed to improve the care of Chagasic patients. The two most common drugs used to treat this condition are benznidazole and nifurtimox, and their respective recommended treatment durations range from 60 to 90 days [9]. Longer treatment sessions place a logistical and financial strain on underprivileged people with limited access to healthcare. Both medications have potentially life-threatening adverse effects, resulting in repeated treatment pauses [14,15,16]. Previously, a study was conducted to determine the effectiveness of a therapeutic DNA vaccine containing *T. cruzi* genes in dogs with experimentally induced Chagas disease [17]. Additionally, because several new vaccination applications have been proposed, most vaccines produced for dogs have been modified [17]. Its value associated with a prophylactic vaccination is clear and supported by several pre-clinical investigations, although specific performance and cost-effectiveness considerations must be addressed [16]. It is unclear, for instance, how findings from studies of vaccine short-term (acute phase) efficacy may be extrapolated to its longer-term (acute phase) effects, which are more likely to have therapeutic value [16]. As a result, a more trustworthy vaccination is required for long-term effectiveness.

The design of vaccines through an immunoinformatic approach against pathogens makes it possible to rapidly discover safe, reliable, inexpensive, and uncomplicated immune responses to the directed antigens. However, an in-vivo and in-vitro experiment is needed to further validate the efficacy of the vaccine candidate. Epitope-based subunit vaccines have been successful in the postgenomic era in stimulating resistance to some of the worst human pathogens, such as influenza, Zika, Ebola, and Middle East respiratory syndrome coronavirus (MERS-CoV) [18,19,20,21,22]. Neither the host nor other species are at risk because subunit vaccines cannot multiply within the host [23,24,25]. However, possible concerns can arise associated with recombinant vaccines including toxicity and allergenicity. Immune responses against specific microbiological determinants can be targeted by subunit vaccines, which can be freeze-dried so the products can be transported and stored without refrigeration. Nevertheless, because the antigen is recombinant, in some cases, the vaccination cannot withstand dehydration or freezing. Other vaccine components, such as alum-based adjuvants, may make it difficult to freeze vaccines. As they are synthetic, mutations or reverses cannot occur, nor are they contaminated with toxins or pathogens. Changing the peptide structure chemically could make the vaccine more stable while reducing its adverse effects [26,27]. In this case, the main goal was to develop a highly antigenic monovalent subunit vaccine candidate against *T. cruzi*, responsible for severe Chagas disease in humans.

## 2. Materials and Methods

**Figure 1:**
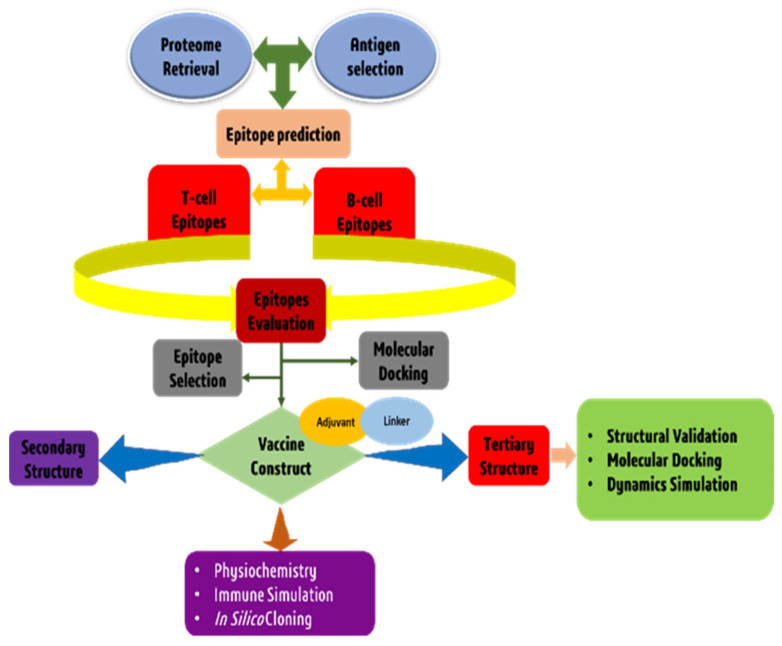
A diagram showing the architectural flow chart of the methodology.

An architectural flow chart of the methodology used in this study is shown in Figure 1.

### 2.1. T. cruzi Core Proteome Identification

The core proteome of the *T. cruzi* genomes was analyzed using a Perl script after retrieving all three sequenced genomes (ID: 31973, 25 and 10993) from the NCBI database [28]. The proteomes were clustered using USEARCH, and proteins having a sequence identity of 50% or less were omitted. As a result of the clustered sequences, all input genomic sequences have been inspected for the presence or absence of proteins. Finally, while developing a vaccine candidate, the sequences of each core protein that is conserved across all proteomes were also considered. These conserved sequences are promising possibilities for developing broad-spectrum antigenic candidates [29].

### 2.2. Subtractive Proteomics Approach

The core proteome is subjected to subtractive proteome analysis to uncover possible novel vaccination targets. The first stage of subtractive proteomics is the elimination of duplicate sequences. Cluster Database at High Identity with Tolerance (CD-HIT) compares and organizes nucleotide or protein sequences to eliminate redundant data and improve the efficacy of sequence analysis. To minimize sequence mismatch, CD-HIT is the most popular program. In this experiment, we utilized the CD-HIT server to filter the entire core proteomics at an 80% confidence level. It aims to decrease recurrence by conforming to a user-defined sequence identity threshold [30]. Proteins retrieved from the CD-HIT service were utilized in a BlastP search for a non-redundant database for non-homologous *T. cruzi* proteins. BlastP (Protein–Protein BLAST) compares the query protein to the database chosen by the user and returns the protein sequences from the other databases most like the query [28]. If query coverage surpassed 70% and identity exceeded 30%, proteins were classified as non-homologous. To create effective vaccines, knowing how a particular protein works are essential. The prediction of the subcellular localization of a protein is an approach that can help to determine the biological function of the protein. Furthermore, investigations have demonstrated that, because proteins are confined in numerous places, localization is crucial in creating vaccine candidates. The subcellular distribution of non-homologous proteins was predicted using the CELLO platform [31]. Virulence proteins are vital because of their significant role in the disease’s pathogenesis. The VFDB (Virulence Factor Database) was used to determine whether a protein had a non-homologous sequence [32]. *T. cruzi* homologs with bit scores greater than 100 and identities more than 30% were termed virulent. Transmembrane helices were determined using a TMHMM server. A program called TMHMM (Transmembrane Helices; Hidden Markov Model) can determine whether a protein contains transmembrane helices or not [33]. Proteins with a significant proportion of transmembrane helices were excluded from the study as they are challenging to synthesize, refine, and clone, making them unsuitable for vaccine development [34]. We prioritized the most antigenic proteins that lack transmembrane helices for vaccine development. Later, the Vaxijen server was used to assess the antigenicity of pathogenic proteins [35]. The antigenic proteins with the highest possible ratings for their antigenicity were selected as potential vaccination candidates. In addition, the allergenicity of the proteins was evaluated by the AllerTOP program, and the Protparam tool was utilized to determine the molecular properties of the proteins [36,37].

### 2.3. Epitopes Prediction and Assessment

#### 2.3.1. Cytotoxic T-Cell Lymphocytes (CTLs)

Several immune system cells, including CTLs, can directly eliminate other infectious cells [38] and directly begin attacking the pathogen’s cell after being ingested. The MHC-I epitope prediction server used only the frequent components to predict highly immunogenic T-cell epitopes [39]. The validity of the anticipated epitopes was confirmed by the VaxiJen v2.0 [35], MHC class I immunogenicity [39], ToxinPred [40], and AllerTop v2.0 [41] servers. Each server’s default settings were used to generate all forecasts.

#### 2.3.2. Helper T-Lymphocytes (HTLs)

HTLs are an example of adaptive immunity because they identify foreign antigens and activate B and cytotoxic T cells, eventually eradicating the infectious pathogen [42]. The MHC class II binding allele predicting tool that can be found on the Immune Epitope Database (IEDB) was utilized to establish HTL epitopes. The CONSENSUS technique was used to choose the HTL epitopes, and a percentile rank of 5% was used [43]. The anticipated epitopes were studied further for their antigenicity and ability to induce cytokines, particularly IFN gamma, IL4, and IL10. The VaxiJen v2.0 server was used to predict antigenicity, and IFN gamma and IL4, and IL10 characteristics were predicted using IFNepitope [44], IL4pred [44], and IL10pred [45] servers, respectively, with default parameters.

#### 2.3.3. Linear B-Lymphocytes (LBLs)

It is necessary to have B-cell epitopes to improve humoral or antibody-mediated immunity. Amino acid subunits compose B cells, which connect to and activate antibodies produced by the immune system to fight off diseases [46]. To do this, we utilized the iBCE-EL server’s default settings to make predictions for linear B-lymphocyte (LBL) epitopes [47]. Common epitopes were tested using VaxiJen v2.0, ToxinPred, and AllerTop v2.0.

### 2.4. T-Cell Epitope Population Coverage and Conservation Analysis

Analysis of population coverage for each epitope was performed using the IEDB population coverage computation tool and resource. Conservation analysis is a powerful tool for finding the degree of homology between homologous proteins. To assess how well-conserved critical epitopes are between species, we used the Epitope Conservancy Analysis Tool from the IEDB.

### 2.5. Modeling of Peptides and Molecular Docking

The PEP-FOLD v3.0 server was used to run simulated experiments on the chosen CTL and HTL epitopes. To accomplish the objective of predicting the 3D structure of the selected peptides, the sOPEP sorting system with 200 simulations was employed [48]. Using sOPEP as a key to sort through the clusters will often result in proposing native or near-native conformations in the top 5 ranks of the peptide model. The Protein Data Bank (PDB) was used to retrieve the crystal structures of selected HLA alleles [49] and modified with BIOVIA Discovery Studio 2017 for docking analysis. Molecular docking was performed using the program AutoDock to generate a square around the binding site of each HLA allele. Molecular docking between epitopes and their associated HLA alleles was also performed using the AutoDock Vina script [50]. To evaluate the relative efficiency of epitope binding, we employed the appropriate co-crystal ligands as a positive control. The docked complex was visualized using BIOVIA Discovery Studio 2017 and PBDSum.

### 2.6. Construction of a Multi-Epitope Vaccine Candidate

The epitope cluster analysis tool in IEDB was used with a sequence identity threshold of 100% to identify conserved peptides among the most potent CTL, HTL, and B-cell epitopes. Vaccine molecules were constructed by using and recombining clusters and individual epitopes. The top epitopes were placed first in each design, followed by an adjuvant. Adjuvant interactions with toll-like receptors (TLRs) cause solid immunological responses in the host [51]. Assembling the vaccine sequence required combining the chosen epitopes with an efficient adjuvant and linking them with the suitable linkers [52,53]. Since viral glycoproteins and adjuvants that recognize TLR4 are required to circumvent translation and synthesis limitations, a TLR4 agonist was used as an adjuvant [54,55]. 50S ribosomal protein L7/L12 (NCBI ID: P9WHE3), an adjuvant, was thus investigated to determine if it may enhance the designed vaccine immunogenicity. The EAAAK bi-functional linker, which can dissociate two b domains from weakly interacting contacts over a broad peptide length range, was used to connect the adjuvant to the vaccination candidate front. In contrast, the selected CTL was linked using AAY linkers, the HTL was linked using GPGPG linkers, and the LBL was linked using KK linkers [42,52]. Proteasome cleavage at the AAY linker has been used to modify protein stability, immunogenicity, and epitope presentation [56]. By employing GPGPG, the immune system can deal with the vaccine sequence, and the bi-lysine KK linker keeps the vaccine’s individual immunogenic characteristics intact. While the GPGPG linkers may prevent junctional epitope development and enhance immune processing and presentation, the EAAAK linkers enable domain partitioning in bifunctional fusion proteins [57]. The AAY linker is also commonly employed in an in silico vaccine design due to its known ability to form effective epitope conjugation.

### 2.7. Structural Analysis of Multi Epitope Reverse Vaccine (MERV) Construct

The physiochemistry of a protein describes its essential properties. To fully understand the vaccination’s crucial role, the ProtParam server made predictions about its physicochemical features [58]. VaxiJen v2.0 [35] was used to predict antigenicity, MHC-I for immunogenicity [39], and AllerTop [41] servers were used to predict allergenicity. The 2D structural features of the designed vaccine were analyzed by SOPMA and PSIPRED v4.0 server. More than 80% of the time, SOPMA can make correct forecasts [59]. The effectiveness of the recently developed vaccine was evaluated by drawing on structural factors from its two-dimensional representation. It was shown that extracting and analyzing 2D structural data provided a better understanding of the vaccine candidate’s composition quality. Solubility was determined by analyzing the suggested surface charge, hydrophobicity, and stability of the vaccine at 91 different pH and ionic strength combinations using the program Protein–Sol [60].

### 2.8. 3D Structure Prediction and Confirmation

The 3D structure of the vaccine sequence was predicted using the RaptorX server. The RaptorX server uses cutting-edge algorithms and 3D structures to generate the most accurate protein structures and their effects [61]. This server provided the five best models of any protein sequence based on TM score, RMSD value, and C score. The PDB file containing the resulting 3D structure was chosen based on the C-score value. The value ranges from −5 to 2 represents the assessment score of the server, and usually, the best 3D model has the highest score according to the server. To refine the best 3D model, the Galaxy Refine server was used, an accessible web-based server. An initiator-type caspase approach called CASP10 was applied to operate the webserver [62]. On the Galaxy Refine website, users can get the RMSD value of the model as well as the energy value and final quality score. Using the minimum and maximum RMSD scores of the optimized structure, we selected the one with the best energy efficiency. To demonstrate the modified structure, PyMOL v2.3.4 was utilized. The Ramachandran plot analysis and Z-score value can calculate the mean and standard deviations by analyzing the vaccine’s final 3D structure. PROCHECK, an application that checks the most allowed and disallowed regions of protein sequences, analyzed the Ramachandran plots, and ProSA-web analyzed the Z-score plots [63].

### 2.9. Discontinuous B Cell Epitope Prediction

More than 90% of B-cell epitopes were discovered to be discontinuous and apart. Discontinuous (conformational) B-cell epitopes have been modeled in 3-space using the computer tool ElliPro. ElliPro produces three methods based on the protrusion index (PI) values for defining the protein form as an ellipsoid, the residue PI, and nearby cluster residues. For each output epitope, ElliPro determines its mean PI value. This value represents the total of the residues that make up each epitope. Ninety percent of the protein residues in the ellipsoid with a PI of 0.9 are inside, whereas only ten percent lie outside. Each epitope residue’s PI was determined by its location outside a maximal ellipsoid of residue mass. ElliPro is the most excellent structure-based technique for predicting epitopes we have discovered, with an AUC of (0.837), the highest of any protein prediction method. Furthermore, using the IFNepitope with motif and SVM hybrid prediction techniques, IFN-inducing epitopes inside the vaccine candidate were predicted [64].

### 2.10. Disulfide Engineering for Vaccine Candidate

The developed model must be stable enough to proceed and begin docking analysis. Disulfide-bonded proteins have a geometrically stable structure. Disulfide by Design 2.0 [65] was used to assign such bonds for the targeted vaccine. All Disulfide by Design (DbD) analysis settings are configured in the server’s Options Pane. DbD tests all probable inter-and intra-chain disulfides by default. Uncheck Intra-chain to limit your investigation to only probable bonds between chains. The DbD method requires coordinates for Cβ atoms to assess the possibility of disulfide formation. The Cβ-Sγ-Sγ-Cβ bonds produce the 3-torsion angle, which rotates around the Sγ-Sγ bond. Disulfides of known protein structures have a bimodal distribution of 3 angles, with prominent maxima at +97° and −87°. The drop-down list of values can be used to enhance or reduce the 3-angle tolerance. +97° ± 30° and −87° ± 30° are the default settings. Engineered disulfides have improved protein stability, allowing researchers to understand better protein dynamics and interactions [66,67,68].

### 2.11. Molecular Docking

Research on molecular docking can give insight into the interactions between modeling proteins and receptor molecules. For molecular docking, we utilized the HADDOCK server version 2.4 to submit the improved vaccination candidate model as a ligand, and the MHC I (PDB Id: 1I1Y), TLR4 (PDB Id: 4G8A), and MHC II (PDB Id: 1KG0) proteins as immunological receptors [69]. The TLR4 immune receptor was selected for docking with the immunogenic construct because it can trigger cytokine overproduction and upregulate the TSPO-associated protein, whereas antigen presentation by major histocompatibility complex (MHC) proteins is essential for adaptive immunity. HADDOCK uses dynamic docking to create biomolecular complexes based on available data. Separating the related ligand from the protein was the first step in preparing the receptor, followed by removing water and other contaminants. HADDOCK distinguishes itself from ab initio docking methods by the fact that it encodes information from identified or predicted protein interfaces in ambiguous interaction restraints (AIRs) to drive the docking process. It also allows the definition of specific unambiguous distance restraints (e.g., from MS cross-links) and supports a variety of other experimental data, including NMR residual dipolar couplings, pseudo contact shifts, and cryo-EM maps. The PyMOL v2.3.4 software was used for these experiments [70]. Later, we utilized Discovery Studio 2017 and PBDSum to examine binding interactions and surface residues.

### 2.12. MD Simulation and Analysis in Normal Mode

Candidates for binding to the active site cavity of the target protein have been studied using molecular dynamic simulations (MDS) [71]. The MDS was run in Schrödinger 2020-3 using the ‘Desmond v6.3 Program’ to analyze the receptor–ligand complex’s thermodynamic stability. The system was solved using a predefined TIP3P water model, and the irregular boundary box shape was orthorhombic with ten on both sides to preserve a constant volume. After combining a protein and a ligand, the system was minimized and relaxed by the default method after the solvated system was produced. To assess the stability of the vaccine complex, the simulations were run for 50 ns, and the trajectory RMSF, RMSD, and secondary structure of proteins were analyzed. Normal mode analysis (NMA) was carried out to enhance the prediction on the iMODS server [72]. Due to significantly quicker and more effective evaluations than alternative molecular dynamics (MD) simulation techniques, the structural dynamics of the protein-TLR4 complex were studied [73]. The server analyzed the normal modes (NMA) in internal coordinates to describe the collective motion of proteins. To assess protein stability, the fundamental dynamics of proteins were linked to their usual modes [74]. The eigenvalue, motion flexibility, elastic network model, and covariance matrix were also examined.

### 2.13. Modeling the Immune System

The whole construct was uploaded to the C-IMMSIM v10.1 server to assess the designed vaccine’s immunological response [75]. The previously recommended minimum interval of 30 days between doses was employed [75]. This study used three in silico injections, with time steps of 1, 84, and 168 (Each time step equaled 8 h in real life). The maximum simulation step value was 500, and all other stimulation parameters were kept at normal ranges.

### 2.14. Codon Optimization and In-Silico Cloning

In any host organism, the expression of a DNA segment necessitates codon optimization [76]. The vaccine reverse transcriptase sequence was then submitted to the JCat service for codon optimization. In this study, an *E. coli* K12 host was utilized, and the entire process was designed to avoid ribosome binding sites, cleavage sites, and termination of rho-independent transcription. The changed sequence’s CAI value and GC concentration were utilized to assess it [60]. The modified nucleotide sequence was then utilized for cloning the expression vector pET28a (+) in silico. The complete in silico cloning analysis was carried out using SnapGene v4.2 [77]. The translation efficiency and thermodynamic stability of the expressed mRNA sequences were also calculated using the RNAfold service [78].

## 3. Results

### 3.1. Examination of the Core Proteome

Because these core proteins are found in most target pathogen strains, using them in vaccine formulations provides immune protection against a larger spectrum of infections. When developing vaccines to combat *T. cruzi*, three primary strains of the disease have been considered. After examining the core proteome, the total number of proteins found in these strains was lowered to 7753 from the previous total of 40,294.

### 3.2. Identification of Interest Proteins

The core proteome of *T. cruzi* was investigated using subtractive proteomic analysis, which used several computational methods and databases. The core proteome is made up of 7753 proteins in total. From 7753 proteins, 227 proteins were recovered using CD-HIT at an 80% threshold by removing paralog sequences. Thus, non-redundant proteins that are not necessary for survival could not be the primary targets of any given attack. Since these proteins are needed for pathogen survival, finding essential proteins distinct from host proteins is crucial for avoiding therapeutic cross-reactivity [79]. Using BlastP, a non-homologous essential protein was discovered. Two hundred and twenty-seven proteins with homology to humans lower than ≤30% were found using BlastP; 103 were essential. Protein functions were characterized by employing a subcellular localization prediction model. Seven of the expected 103 cytoplasmic targets were left out of the experiments. The remaining 96 proteins were evaluated against the VFDB; 17 virulent proteins were identified with a bit score larger than >100 and a sequence identity greater than ≤30%. These proteins were distributed among 36 extracellular, 14 plasma membrane, 29 mitochondrial, and 17 periplasmic locations. Their antigenicity was predicted using VaxiJen v2.0. From a total of 17 proteins, four were shown to be particularly allergenic. Furthermore, it was discovered that four of these proteins lacked transmembrane helices. Moreover, they were shown to have molecular weights up to 50 kDa, making them ideal targets and prospects for vaccine development because they are not allergenic. Table 1 provides a comprehensive catalog of the proteins.

### 3.3. Prediction of Epitopes

In selected target proteins, our experiments were run to look for CTL, HTL, and LBL epitopes. There were 113 unique CTL epitopes predicted with MHC-1 binding alleles in total. A list of the top CTL epitopes with immunogenic features was compiled (Table 2).

The IL-4 and IL-10 inducers and IFN-γ-positive HTL epitopes selected for multi-epitopes-based vaccine design were selected from 87 HTL epitopes with MHC-II binding alleles. The top HTL epitopes are shown in Table 3.

The top LBL epitopes for MEBV design were selected based on their toxicity, immunogenicity, antigenicity, and non-allergenicity out of 95 distinct LBL epitopes that were predicted (Table 4). However, no LBL epitopes were predicted from the Ribosomal Protein P1.

### 3.4. T-Cell Epitope Population Coverage and Conservation Analysis

**Figure 2:**
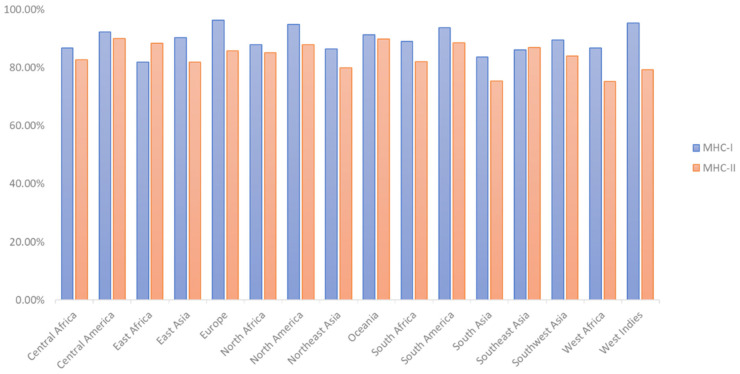
Antigenicity based on MHC-I and MHC-II population coverage study of anticipated T-cell epitopes.

According to the findings, the projected T-cell epitopes can encompass people from all around the world (Figure 2). In various strains, putative epitopes formed from the four proteins were highly conserved (Table 2). Molecular docking research was conducted on highly conserved epitopes likely to elicit a comprehensive immunological response.

### 3.5. Epitope and Allele Docking Studies

A PEPFOLD server was used to convert 16 T-cell epitopes (8 CTL and 8 HTL) into 3D structures, and their interactions with HLA molecules were investigated. The top epitopes were docked against class-I and class-II alleles [80]. According to the findings, all the anticipated epitopes are bound in the groove of MHC molecules with suitable binding energy (Table 5).

### 3.6. The Core Properties and Structure of the Vaccine Candidate

**Figure 3:**
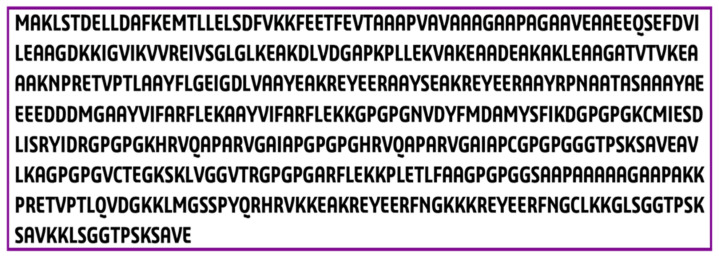
Constructed vaccine sequence.

To create the final vaccine sequence, 22 peptides from three distinct classes (CTL, HTL, and LBL) were employed. Epitopes were linked using AAY, GPGPG, and KK linkers. An adjuvant was applied before the construct to increase immunogenicity. Using the ribosomal protein, and with the 50S/L12 as an agonist, an adjuvant was bound to the CTL epitope via the EAAAK linker to activate TLR4. The final vaccine candidate was 475 amino acids in length (Figure 3).

### 3.7. Immunological Evaluation and Physicochemical Properties

**Figure 4:**
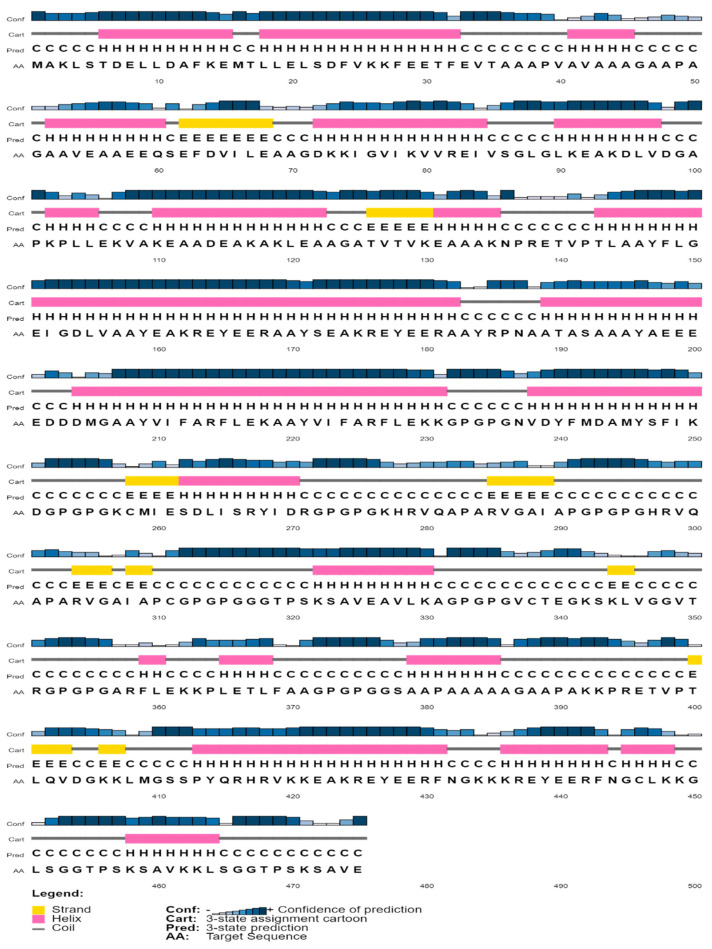
Prediction of the secondary structure of designed multi-epitope vaccines using PSI-PRED.

The molecular weight of the final vaccine candidate was determined to be 49,607.54 Da. Other characteristics included the theoretical pI of 5.29, chemical formula C_2201_H_3525_N_609_O_671_S_11_, instability index of 27.83, aliphatic index of 71.89, and GRAVY of −0.352. The vaccine’s physicochemical qualities and immunological functions were also evaluated. Vaccines, for instance, exhibited antigenicity of 0.6635 and immunogenicity of 1.08175. Furthermore, the vaccine was soluble and non-allergenic (Table 6). Secondary structure features such as helices, beta strands, and coils were probed by employing two separate servers. 50.74% of the construct was projected to be α helices, 7.16 % was anticipated to be β strands, and 30.53% was predicted to be random coils (Table 6). Also, the PSIPRED server predicted the secondary features of the final vaccine sequence as 48.84% α-helix, 7.157% β-strand, and 44% random coils (Table 7) (Figure 4).

### 3.8. 3D Structure Refinement

**Figure 5:**
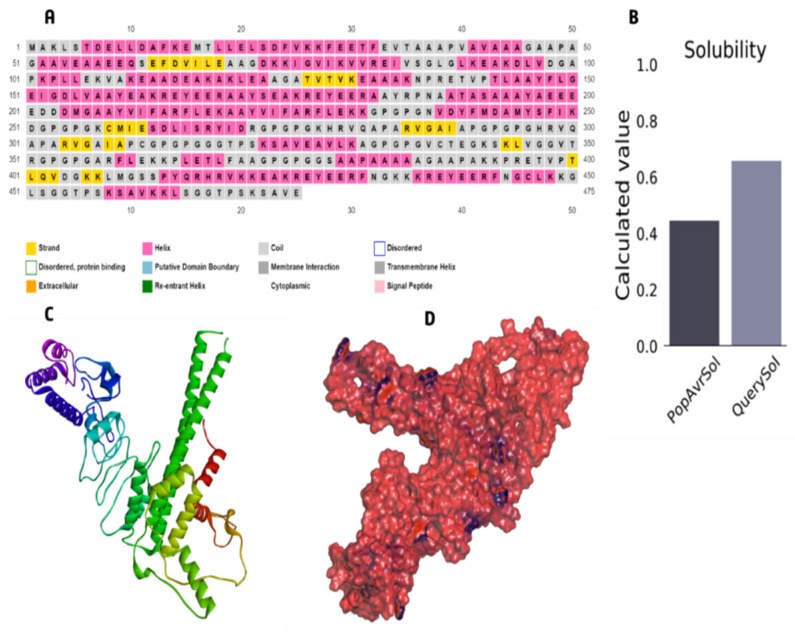
(**A**) 2D structure analysis, (**B**) vaccine build solubility prediction shows that the soluble *T. cruzi* protein from the experimental solubility dataset is more soluble than average. 3D structure of vaccine protein via RaptorX (**C**) Cartoon format, (**D**) Surface structure.

**Figure 6:**
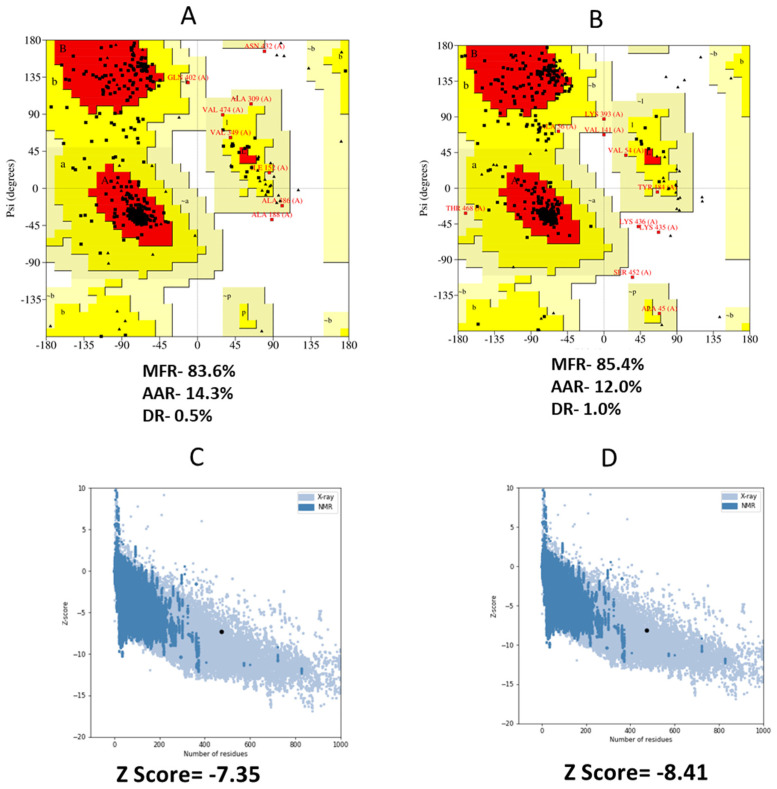
(**A**,**B**) PROCHECK server analysis of Ramachandran plot. The most preferred, additionally allowed, generously allowed, and disallowed areas of the vaccination were represented as the Most Favored Region (MFR), Additional Allowed Region (AAR), Generously Allowed Region (GAR), and Disallowed region (DR). (**C**,**D**) 3-D structure validation with a Z-score by the Pro-SA server.

The best starting point for developing the top five homology models was the RaptorX template. Five different models were considered, and we ultimately settled on the one with the lowest C score (−4.90). Figure 5 shows a 3D depiction of the vaccination with the predicted solubility. In the Ramachandran plot, the vaccine’s most favorable zone was 83.6%, the additionally allowed zone was 14.3%, and the disallowed zone was 0.5% before refinement. Moreover, following refinement, the Ramachandran plot revealed 85.4% of the residues in the most favorable zone, 12.0% in additional allowed regions, and 1.0% in prohibited regions (Figure 6A,B). The final Z score was −8.41 in the refined model (Figure 6C,D).

### 3.9. Conformational B Cell Epitopes Prediction

**Figure 7:**
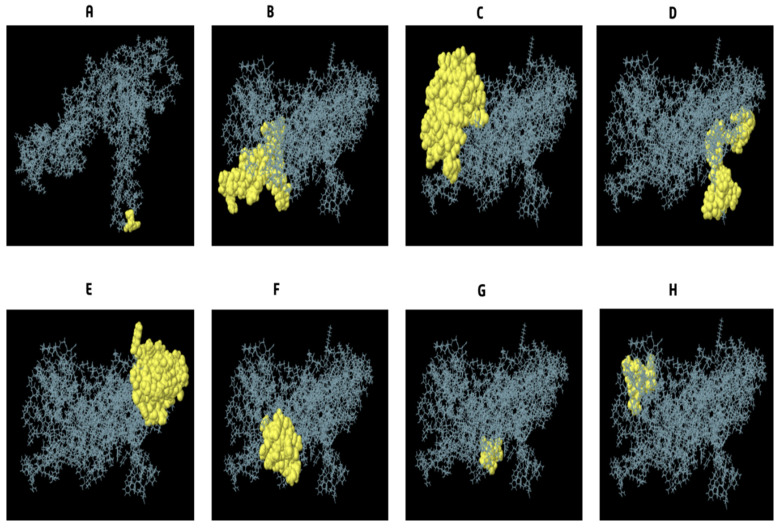
Dimensional display of conformational or discontinuous B-cell epitopes used in the epitope-based vaccine design. (**A**–**H**) Yellow surfaces show conformational or discontinuous B-cell epitopes, whereas grey sticks depict the rest of the polyprotein.

We calculated that there are 245 different residues over eight different discontinuous B-cell epitopes, with values from 0.53 to 0.994. Different conformation epitopes ranged in size from 3 to 67 amino acids. For the predicted discontinuous peptides using Ellipro, the cutoff score was set at 0.53 (Figure 7A–H) and (Table 8).

### 3.10. Disulfide Engineering for Vaccines

The vaccine design was stabilized with the use of disulfide engineering. In the instance of our vaccine candidate, the DbD2 server found 30 possible pairings of amino acids that may create disulfide bonds. After factoring in things like energy and the chi3 value, two sets of cysteine mutations were suggested. As a result, the residue pairings with the most alterations were SER67-CYS185 and ALA147-CYS206. The allowed values for energy and chi3 are less than 4.81 and 110.75: −105.33, respectively.

### 3.11. Molecular Docking Research

**Figure 8:**
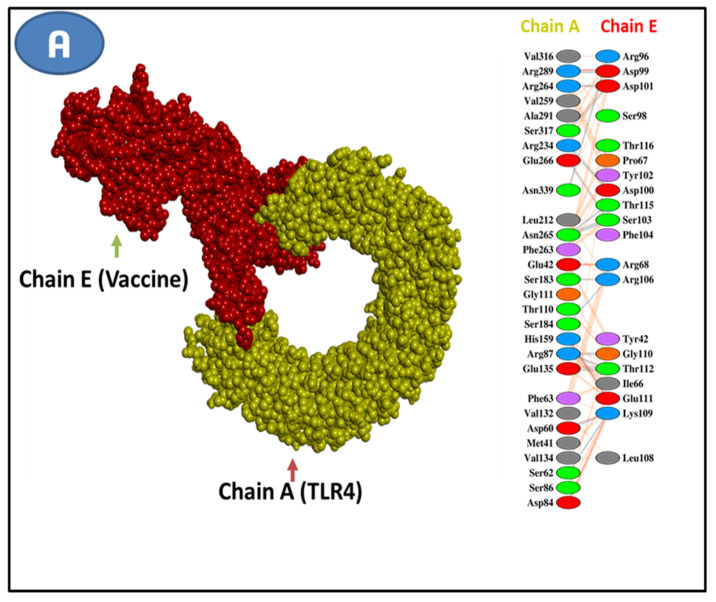

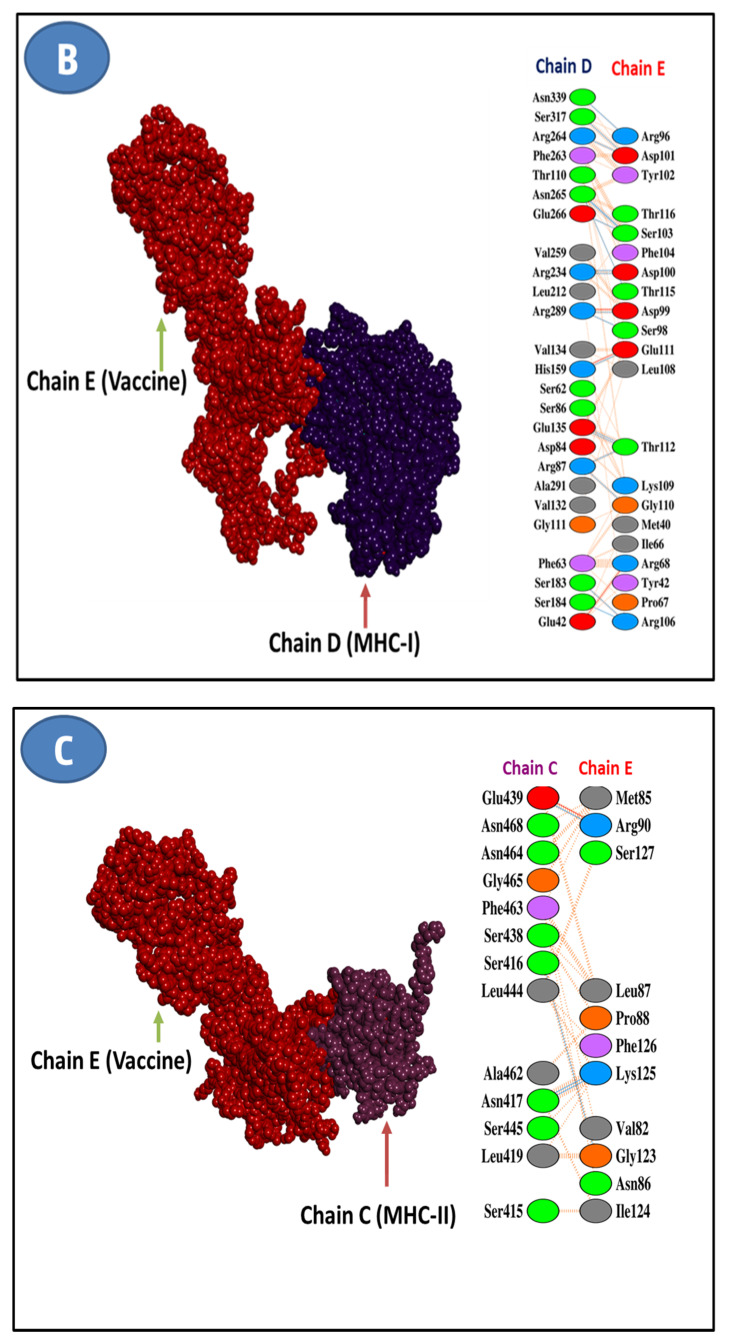
Analysis of vaccine–receptor binding conformation and interaction with (**A**) Vaccine–TLR4, (**B**) Vaccine–MHC I, and (**C**) Vaccine–MHC II.

TLR4, MHC I, and MHC II receptors were docked with the vaccine sequence as the ligand to anticipate their binding affinity and interactions. Using this strategy, we could generate ten docked complexes on the HADDOCK server. From among the complexes, the one with the lowest energy score and the binding posture, including functional connections, was selected. The energy scores of the docked ligand and receptor complexes led us to select model 1 as the best fit for all the complexes. Vaccine-TLR4, Vaccine-MHC I, and Vaccine-MHC II all had energy scores of 986.5, 961.5, and 899.5, respectively. The HADDOCK server predictions for docking with these three complexes are also included in Table 9 below. The PDBsum website analyzed the chosen chemical for vaccine-binding interactions and active site residues. The vaccine–TLR4 receptor combination was discovered to have fifteen hydrogen bonds on its contact surface. There were 13 different kinds of classical hydrogen bonds among them (Figure 8A). A total of 15 hydrogen bonds were identified in vaccine–MHC I complex, 11 of which were classical (Figure 8B). However, we found only three hydrogen bonds in the vaccine–MHC II complexes (Figure 8C).

### 3.12. MD Simulation

**Figure 9:**
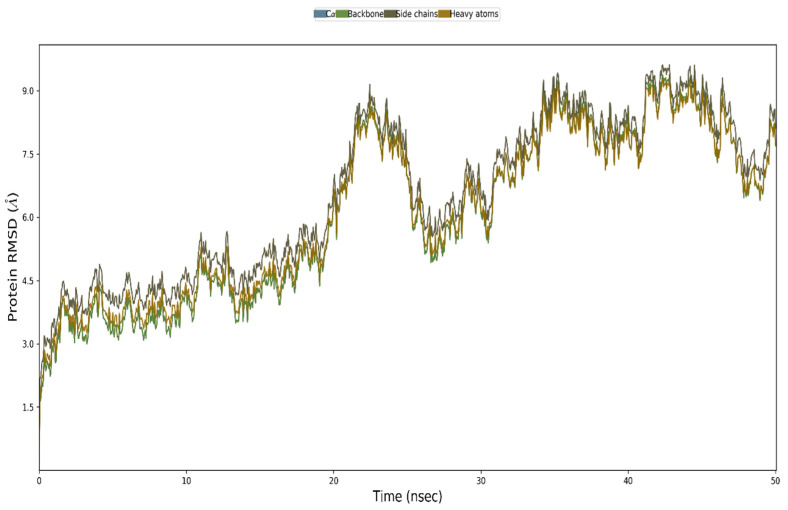
Modeling the multi-epitope vaccine complex at the molecular level. The backbone atoms of the complexes were seen using the RMSD method.

**Figure 10:**
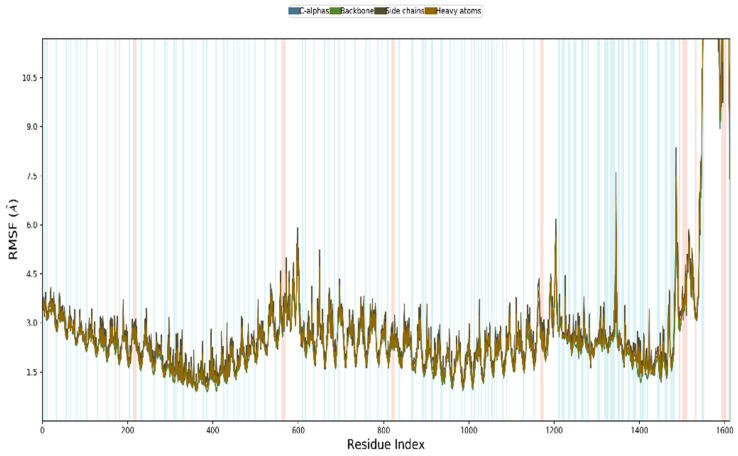
The multi-epitope vaccine complex is simulated. A multi-epitope docked vaccine candidate is shown in the RMSF figure. Red and blue backgrounds, respectively, emphasize the alpha-helical and beta-strand portions. These places are identified by helixes or strands that last for 70% of the simulation time.

**Figure 11:**
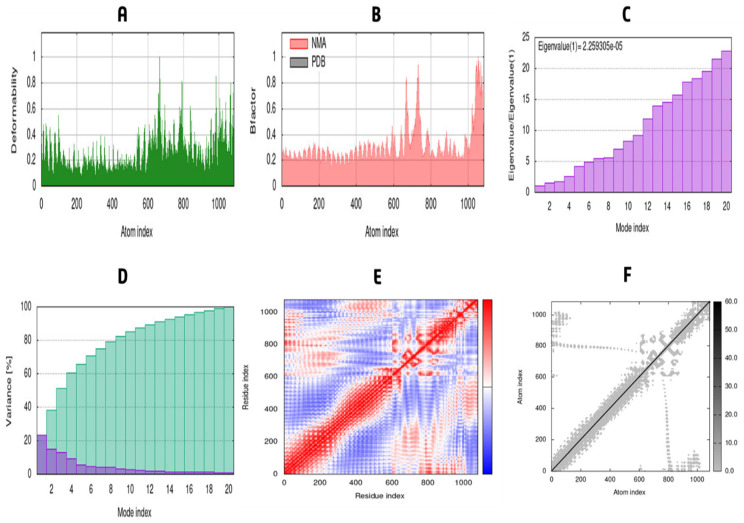
Normal mode analysis of Vaccine-TLR4 complex. The protein-protein complex’s stability was studied using deformability (**A**), B-factor (**B**), eigenvalue (**C**), variance (**D**), covariance (**E**), and elastic network (**F**) analysis.

The vaccine complexes and root-mean-square deviation (RMSD) were calculated. The vaccine complex’s average RMSD value was 4.74 Å, indicating structural stability during the interaction. This figure shows that the RMSD characteristics of the vaccination complex increased rapidly until eight ns, after which they remained stable until 12 ns. This stability and the ability to form strong bonds may result from a drop in values from 20 to 28 ns (Figure 9). Root-mean-square fluctuation (RMSF) was also used to measure amino acid residue-to-residue protein flexibility. According to the RMSF profile of the vaccine complex, the most abundant amino acid residues are from complexes with an RMSF below 4.0 Å, and more significant changes were seen for a smaller number of residues. The vaccine complex’s stability and stiffness are shown in Figure 10. Normal mode analysis was utilized to characterize protein complex stability and global mobility. The placement of hinges in the chain (as indicated in Figure 11A was insignificant, but the B-factor column provided an average of RMS (Figure 11B). The calculated higher eigenvalue (2.259305 e) indicated that the protein complex was unlikely to deform (Figure 11C). Individual (blue) and cumulative (green) variances were negatively linked to eigenvalues in Figure 11D. As seen by the red, white, and blue values in the covariance matrix, correlated, uncorrelated, and anti-correlated motions are depicted in the matrix (Figure 11E), highlighting the coupling between residues. In contrast, the elastic network model (Figure 11F) identified the pairs of atoms connected by springs (the darker the grays, the stiffer the springs).

### 3.13. Simulation of Immune Response

**Figure 12:**
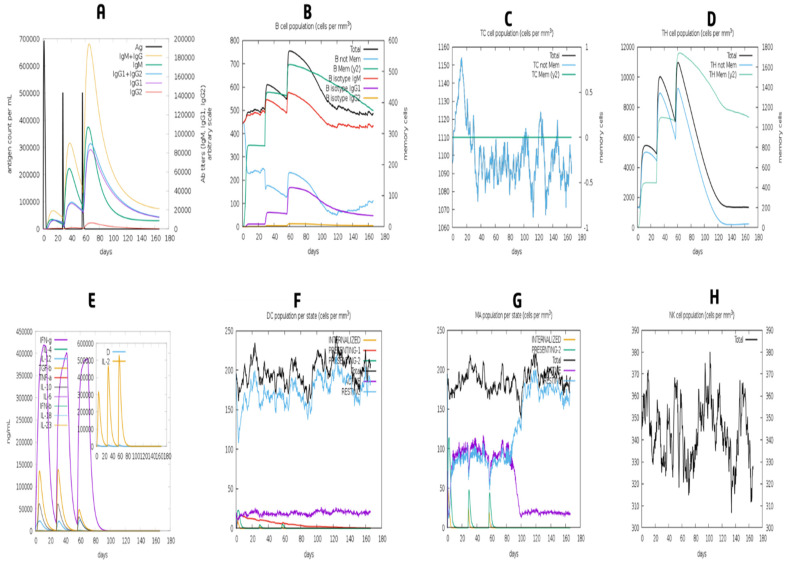
The vaccine has elicited an immune response. The graph shows (**A**) immune responses of vaccine, (**B**) B-cell population, (**C**) cytotoxic T-cell population, (**D**) helper T-cell population, (**E**) induction of cytokines and interleukins, (**F**) dendritic cell population per state, (**G**) macrophage population per state and (**H**) Natural Killer cells (total count).

As can be shown in Figure 12, the model immune response came close to the real-world immune responses triggered by various diseases. Primary immune responses, for example, were higher than secondary and tertiary immune responses (Figure 12A). Further exposures elicited secondary and tertiary responses, which were associated with elevated antibody levels and dramatically improved antigen clearance (Figure 12A). IgM memory development and immune cell class flipping were also evidenced by the extended survival of B cells, cytotoxic T cells, and helper T cells (Figure 12B–D)). The presentation showed increased macrophage mobility and natural killer cells, although dendritic cell movement was expected (Figure 12F–H)).

### 3.14. Codon Adaptation and In Silico Cloning

**Figure 13:**
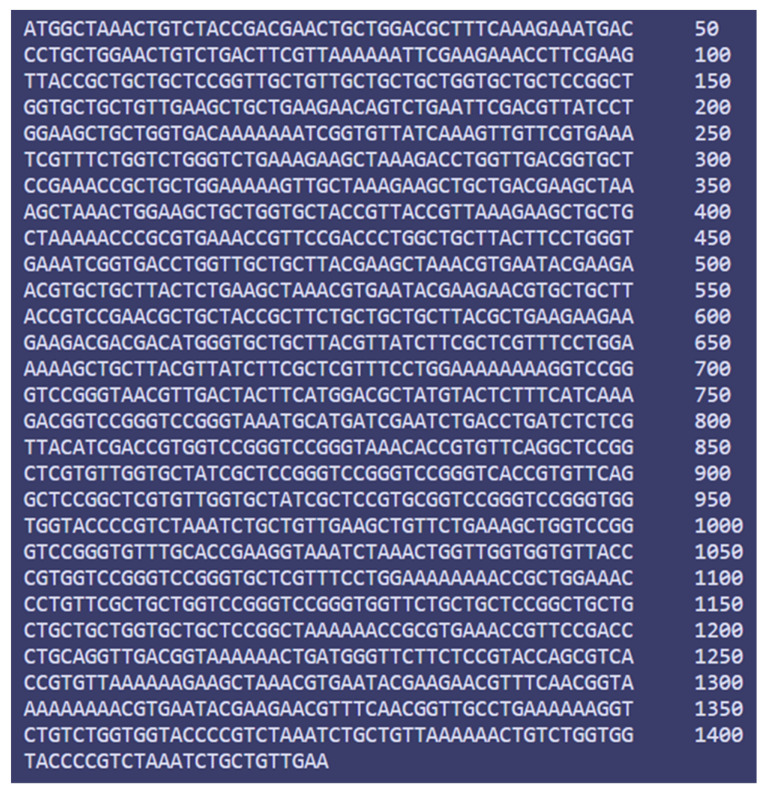
Codon adaptation of EBV to *E. coli* K12 strain.

**Figure 14:**
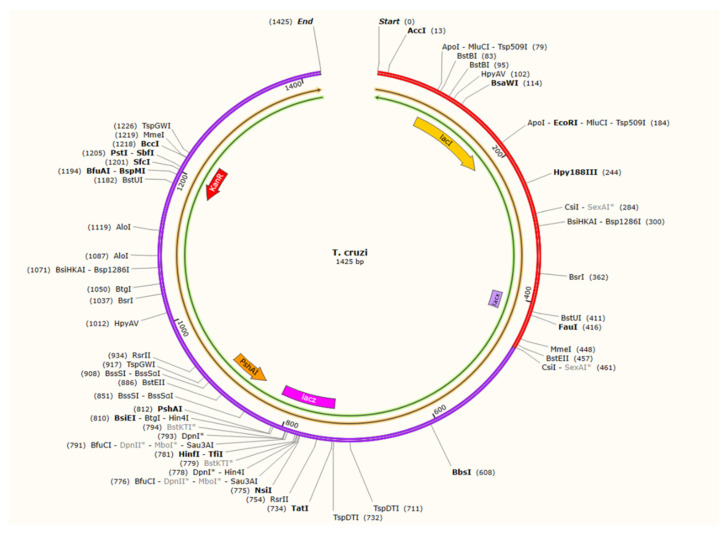
The vaccine was cloned into the pET−28a (+) vector.

**Figure 15:**
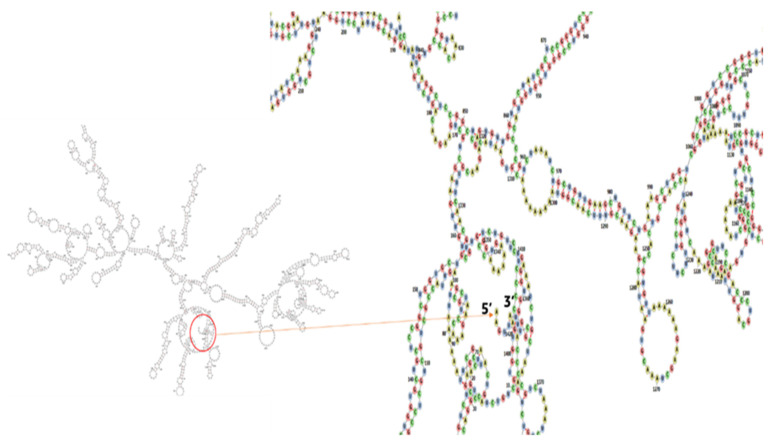
The vaccine’s predicted secondary structure of mRNA. There are no pseudoknots or hairpins at the 5′ end of the projected mRNA structure.

To improve the translation efficiency of the vaccine design, we used the JCat service to adapt the codons to the *E. coli* K12 strain. The nucleotide sequences produced by the peptide vaccine construct (475 AA residues) were 1425 bases in length (Figure 13). Results from the further analysis showed that the modified nucleotide sequence had a GC content of 51.64 percent and a CAI value of 1.0. To insert the modified sequence into the pET28a (+) vector, we employed the pSHAI and BccI restriction sites as start and stop points, respectively. Using SnapGene, the revised vaccine design was cloned into the expression vector pET28a (+) (Figure 14). The RNA fold server was used to make predictions about the secondary structure of messenger RNA. The mRNA structure is thermodynamically stable, as indicated by the minimal free energy of −451.10 kcal/mol. In addition, there were no pseudoknots or lengthy stable hairpins in the first six nucleotides of the mRNA secondary structure, allowing for efficient translation initiation from the mRNA framework (Figure 15).

## 4. Discussion

The present diabolical rise of infections from *T. cruzi* poses the global threat of Chagas disease, prompting us to develop this multi-epitope vaccine using immunoinformatics technology. The vaccine based on pathogenic proteins showed relevance, as expected by immunoinformatics, demonstrating the validity of our efforts. Based on core proteomics, the proposed vaccine showed great relevance, which immunoinformatics had predicted would be the case. This proved that our efforts were valid. Vaccination is a practical and safe method of preventing infectious diseases [81]. It should be possible to do so to develop immunity against infectious illnesses [82]. As a result of our study, we created an epitope-based vaccine to boost the immune system’s defense against Chagas disease. Furthermore, vaccination has not yet effectively controlled the existing human Chagas disease infection situation. The main aim of the study was to design an epitope-based reverse vaccine based on most antigenic and immunogenic epitopes, which is essential for immunological penetration and transmission from species to species. The first step was identifying all possible epitopes of T cells, HTLs, and LBLs through integrated core proteomic analysis of *T. cruzi*. Vaccines were created based on the linkers below matching the top antigenic epitopes that contained T-cell, HTL, and LBL antigenic epitopes. They are essential components of our peptide vaccine to stabilize, fold, and regulate transcription [83]. To stimulate both cellular and humoral immune responses and prevent them from degrading over time, EAAAK linkers were utilized to link the adjuvant with the epitope in the novel vaccine [84]. The vaccine had 475 amino acids in total. Recombinant vaccines must be soluble to be effective [85]. To determine if the vaccine’s design could dissolve inside *E. coli*, a solubility evaluation tool was used, and the results showed that it could. The vaccine was based in nature, as evidenced by the expected PI value. Based on the stability index provided by server tools, the protein should remain stable after synthesis. Based on its physicochemical properties and scores on all parameters, it is expected to be a valid candidate against Chagas disease in humans. Following the prediction of 3D structure, the discovered models were reviewed, and the best model was picked (based on the c score). We discovered a decent number of Z scores (−8.41) and, in many cases, excellent features of most favored, acceptable, and disallowed areas using the Ramachandran plot validation test. In a molecular docking experiment between an epitope vaccine and the viral protein-binding TLR4, the lowest energy score of 986.5 indicates the vaccine would have an infection-inhibitory function and would be tightly bound to the TLR4 receptor [86]. Protein dynamics as a function of time may be modeled to simulate real-world motion. Using a 50 ns dynamic simulation, we measured the RMSD and RMSF to see how well the vaccination candidate performed. RMSD value can compare the different features of molecules in molecular dynamic simulation analysis. In this study, this value offered insight into vaccination candidates’ mobility and the displacement of atoms from the receptor structure in vaccine candidates’ results via RMSF analysis. Calculated RMSDs and RMSFs were 4.74 Å and 4.0 Å, respectively. Normal mode analysis showed the vaccine–TLR4 complex stability. Vaccine and receptor regions showed less variation, but they flattened out within the first half of the MD simulation, suggesting stability. Finally, we used an immunological simulation to examine the optimal parameters of target clearance and cell density for the optimum immune response to the virus. As a result of the higher vaccination dosages, the immune system developed memory B cells (which have a long half-life) and T cells. The vaccination successfully replicated a humoral immune response that enhanced immunoglobulin production. To maximize vaccine output, we used MD simulations to examine the stability of the proposed vaccine candidate with the receptor and codon optimization to ensure the constructed vaccine remains stable within the host throughout the replication of many epitopes. Using the pET28a (+) expression vector for *E. coli* K12, the intended vaccine candidate was cloned successfully.

## 5. Conclusions

Several computational techniques were used in this study to identify potential B- and T-cell epitopes in the *T. cruzi* core proteomics, which were used to stitch together a vaccine based on epitopes. The developed vaccine candidate possesses immunodominant qualities. In response to Chagas disease, it was able to bind to TLR4, MHC-I, and MHC-II and induce a strong immune response in computational analysis. Based on our findings, we believe that designing a vaccine candidate against the agent responsible for the Chagas disease in humans must start with the candidate vaccine. Additionally, the epitopes that may have been discovered in this study can be used in future studies and proof is still needed to confirm the immunogenicity and efficacy of the predicted vaccine. A comprehensive in vivo study is required to prove that our designed vaccine provides complete protection against Chagas disease.

## Figures and Tables

**Table 1:** *T. cruzi* vaccine candidate proteins are described in detail.

Name of Protein	Accession No.	Sub-Cellular Localization	Transmembrane Helices	Antigenicity	Molecular Weight (kDa)
Thiol transferase Tc52	AAO63160.1	Cytoplasmic	0	0.4473	48.12
Ribosomal protein P0	AAA30236.1	Mitochondrial	0	0.4933	34.95
TcP2beta	CAA52941.1	Mitochondrial	0	0.6152	10.57
Ribosomal protein P1	AAT37631.1	Plasma membrane	0	0.6218	11.41

**Table 2:** Final CTL epitopes.

Protein Name	Epitopes	Interacting HLAs Number	Immunogenicity	Allergenicity	Antigenicity	Toxicity	Conservancy (Identity ≤ 100)	Remarks
**Thiol transferase Tc52**	**NPRETVPTL**	**54**	**Positive**	**Non-allergen**	**0.8667**	**Non-toxic**	**100.00% (1/1)**	**Selected**
	RVLITAKEK	27	Positive	Non-allergen	0.8488	Non-toxic	100.00% (1/1)	
	ESQLIVHYL	27	Positive	Non-allergen	0.8358	Non-toxic	100.00% (1/1)	
	**FLGEIGDLV**	**81**	**Positive**	**Non-allergen**	**1.5065**	**Non-toxic**	**100.00% (1/1)**	**Selected**
**Ribosomal protein P0**	SLGAGIPTA	81	Positive	Non-allergen	0.9863	Non-toxic	100.00% (1/1)	
	**EAKREYEER**	**81**	**Positive**	**Non-allergen**	**1.1030**	**Non-toxic**	**100.00% (1/1)**	**Selected**
	YGRVLFCLM	27	Positive	Non-allergen	0.7849	Non-toxic	100.00% (1/1)	
	**SEAKREYEER**	**27**	**Positive**	**Non-allergen**	**1.0618**	**Non-toxic**	**100.00% (1/1)**	**Selected**
**TcP2beta**	**RPNAATASA**	**54**	**Positive**	**Non-allergen**	**1.1933**	**Non-toxic**	**100.00% (1/1)**	**Selected**
	TASAPTAAA	27	Positive	Non-allergen	0.9171	Non-toxic	100.00% (1/1)	
	**AEEEEDDDMG**	**27**	**Positive**	**Non-allergen**	**1.1749**	**Non-toxic**	**100.00% (1/1)**	**Selected**
	EEEDDDMGFG	27	Positive	Non-allergen	0.8471	Non-toxic	100.00% (1/1)	
**Ribosomal Protein P1**	**VIFARFLEK**	**27**	**Positive**	**Non-allergen**	**1.3362**	**Non-toxic**	**100.00% (1/1)**	**Selected**
	LPVIFARFL	27	Positive	Non-allergen	1.2247	Non-toxic	100.00% (1/1)	
	**VIFARFLEKK**	**54**	**Positive**	**Non-allergen**	**1.3986**	**Non-toxic**	**100.00% (1/1)**	**Selected**
	TKEEEEDDDM	27	Positive	Non-allergen	1.1196	Non-toxic	100.00% (1/1)	

**Table 3:** Final HTL epitopes.

Protein Name	Epitopes	No. of Interacting HLAs	IL10	IL4	Antigenicity	IFN-γ	Conservancy (Identity ≤ 100)	Remarks
**Thiol transferase Tc52**	**NVDYFMDAMYSFIKD**	**81**	**Inducer**	**Inducer**	**0.8484**	**Positive**	**100.00% (1/1)**	**Selected**
	SNVDYFMDAMYSFIK	27	Inducer	Inducer	0.5137	Positive	100.00% (1/1)	
	**KCMIESDLISRYIDR**	**27**	**Inducer**	**Inducer**	**0.7380**	**Positive**	**100.00% (1/1)**	**Selected**
	SYHVRFVESNVDYFM	54	Inducer	Inducer	0.5797	Positive	100.00% (1/1)	
**Ribosomal protein P0**	**KHRVQAPARVGAIAP**	**54**	**Inducer**	**Inducer**	**1.1816**	**Positive**	**100.00% (1/1)**	**Selected**
	**HRVQAPARVGAIAPC**	**27**	**Inducer**	**Inducer**	**1.0619**	**Positive**	**100.00% (1/1)**	**Selected**
	PCDVIVPAGNTGMEP	54	Inducer	Inducer	0.6644	Positive	100.00% (1/1)	
	FKTLLGASVATEYEF	27	Inducer	Inducer	0.4773	Positive	100.00% (1/1)	
**TcP2beta**	EGKSKLVGGVTRPNA	54	Inducer	Inducer	0.8056	Positive	98.54% (38/39)	
	VGLSGGTPSKSAVEA	27	Inducer	Inducer	1.2365	Positive	98.54% (38/39)	
	**GGTPSKSAVEAVLKA**	**54**	**Inducer**	**Inducer**	**0.8458**	**Positive**	**100.00% (1/1)**	**Selected**
	**VCTEGKSKLVGGVTR**	**27**	**Inducer**	**Inducer**	**0.8138**	**Positive**	**100.00% (1/1)**	**Selected**
**Ribosomal Protein P1**	EGAAAAPAAGSAAPA	27	Inducer	Inducer	0.8360	Positive	100.00% (1/1)	
	**ARFLEKKPLETLFAA**	**54**	**Inducer**	**Inducer**	**1.1755**	**Positive**	**100.00% (1/1)**	**Selected**
	**GSAAPAAAAAGAAPA**	**27**	**Inducer**	**Inducer**	**0.9713**	**Positive**	**100.00% (1/1)**	**Selected**
	TLPVIFARFLEKKPL	27	Inducer	Inducer	0.7751	Positive	100.00% (1/1)	

**Table 4:** Final LBL epitopes.

Protein Name	Sequence	Score	Antigenicity	Allergenicity	Toxicity	Remarks
**Thiol transferase Tc52**	**PRETVPTLQVDG**	**0.7874**	**1.1616**	**Non-allergen**	**Non-toxic**	**Selected**
	LNPRETVPTLQV	0.7025	0.4951	Non-allergen	Non-toxic	
	SRYIDRISSPAN	0.7828	0.5385	Non-allergen	Non-toxic	
	**LMGSSPYQRHRV**	**0.7522**	**1.0283**	**Non-allergen**	**Non-toxic**	**Selected**
**Ribosomal protein P0**	**EAKREYEERFNG**	**0.8234**	**1.2682**	**Non-allergen**	**Non-toxic**	**Selected**
	SEAKREYEERFN	0.8269	0.7628	Non-allergen	Non-toxic	
	ERFNGCLTKYGR	0.8194	0.7035	Non-allergen	Non-toxic	
	**KREYEERFNGCL**	**0.7704**	**1.5208**	**Non-allergen**	**Non-toxic**	**Selected**
**TcP2beta**	**GLSGGTPSKSAV**	**0.6102**	**1.4848**	**Non-allergen**	**Non-toxic**	**Selected**
	**LSGGTPSKSAVE**	**0.6932**	**1.2704**	**Non-allergen**	**Non-toxic**	**Selected**
	SGGTPSKSAVEA	0.6109	1.1716	Non-allergen	Non-toxic	

**Table 5:** List of residues that generate hydrogen bonds with epitopes, binding affinities, interactions, and docking alleles.

Selected T-Cell Epitopes	PDB IDs of HLAs/Receptors	Epitope Affinity (kcal/mol)	Control Affinity (kcal/mol)	Number of Hydrogens Bonds (CHB)	Residues Involved in CHB Networks
NPRETVPTL	1a6a (HLA-DR3)	−7.3	−6.9	8 (7)	Ala49, Trp7, Ile87, Gly19, Ile11, Ala29, Trp17, Tyr74
FLGEIGDLV	1h15 (HLA-DRA1*0101)	−7.1	−7.0	8 (7)	Thr80, Lys91, Val156, Tyr7, Lys84, Leu66, Thr77, Asn143
EAKREYEER	2q6w (HLA- DRB3*0101)	−7.3	−7.0	9 (7)	Lys26, Asn17, Asn77,Lys89, Tyr84,Tyr99,Thr343, Lys146, Trp34
SEAKREYEER	2seb (HLA-DR4)	−6.9	−7.1	7 (5)	Arg171, Ala12, Asn82, Val1, Glu6, Ser4, Thr77
RPNAATASA	3c5 (HLA- (DRA*0101)	−7.3	−7.5	9 (7)	Tyr17,Asp92,Asp99,Ser241, Lys66, Tyr99, Glu152, Glu152, Gln155
AEEEEDDDMG	2fse (HLA-DRB1*0101)	−6.7	−6.3	7 (6)	Glu80, Trp72, Asn326, Glu7, His145, Phe37, Ile17
VIFARFLEK	1YDP (HLA-G)	−6.9	−6.6	8 (5)	Lys80, Tyr84, Thr146, Val7, Lys9, Val66, Tyr77, Asn143
VIFARFLEKK	2D31 (HLA-G)	−7.7	−7.3	10 (8)	Lys80, Tyr84, Thr146, Val7, Lys9, Val66, Tyr77, Asn143, Val13, Thr14
NVDYFMDAMYSFIKD	3C5J (HLA DR52c)	−7.1	−6.3	9 (5)	Met69, Ile149, Thr7, Asn8, Ala19, Ile1, Gla2, Tyr7, Trp74
KCMIESDLISRYIDR	1EU3 (HLA-E)	−7.0	−6.8	8 (7)	Lys87, Tyr84, Tyr99, Lys149, Thr146, Ile147, Glu152, Glu154
KHRVQAPARVGAIAP	3LQZ (HLA-DP2)	−6.8	−6.0	7 (4)	Ser53, Glu89, Asn72, Ile17, His7, Glu45, Phe17
HRVQAPARVGAIAPC	4GKZ (HA1.7)	−7.0	−6.9	8 (7)	Arg71, Asn12, Ala82, Val17, Ser6, Glu4, Thr77, Thr13
GGTPSKSAVEAVLKA	6J1V (HLA-A*3003/RT313)	−6.7	−6.1	8 (5)	Tyr80, Lys84, Val146, Thr7, Lys9, Val66, Thr77, Asn143
VCTEGKSKLVGGVTR	6J1V (HLA-A*3003/RT313)	−6.5	−6.8	7 (5)	Asn82, Glu1, Glu6, Ser4, Thr79, Ile13, Val114
ARFLEKKPLETLFAA	1KPR (HLA-E)	−7.1	−7.0	7 (4)	Glu85, Trp326, Thr78, Glu45, Phe8, Ile17
GSAAPAAAAAGAAPA	6Z9V(A02 allele)	−6.9	−6.0	7 (5)	Lys146, Trp147, Glu15, Glu152, Tyr84, Tyr99, Thr143,

**Table 6:** Antigenicity, allergy, and physicochemical features of the construct.

Characteristics	Finding	Remark
Number of amino acids	475	Suitable
Molecular weight	49,607.54	Suitable
Theoretical pI	8.53	Base
Chemical formula	C_2201_H_3525_N_609_O_671_S_11_	-
Instability index of vaccine	27.83	Stable
Aliphatic index of vaccine	71.89	Thermostable
Grand average of hydropathicity (GRAVY)	−0.352	Hydrophilic
Antigenicity	0.6635	Antigenic
Immunogenicity	1.08175	Immunogenic
Allergenicity	No	Non-allergen
Solubility	0.658	Soluble

**Table 7:** The vaccine candidate’s secondary structural characteristics.

Characters	SOPMA		PSIPRED Server	
	AA	%	AA	%
α helix	241	50.74	232	48.84
β strand	34	7.16	34	7.157
Random coil	145	30.53	209	44

**Table 8:** The conformational B-cell epitope residues of the planned epitope-based vaccination were predicted using ElliPro.

No.	Residues	Number of Residues	Score
1	A: A182, A:A183, A:Y184	3	0.994
2	A:M1, A:A2, A:K3, A:E164, A:Y165, A:E166, A:E167, A:R168, A:A169, A:A170, A:Y171, A:S172, A:E173, A:A174, A:K175, A:R176, A:E177, A:Y178, A:E179, A:E180, A:R181, A:R185, A:P186, A:N187, A:A188, A:A189, A:T190, A:A191, A:S192, A:A193, A:A194, A:A195, A:Y196, A:A197, A:E198, A:E199, A:E200, A:E201, A:D203, A:D204	40	0.833
3	A:K393, A:P394, A:R395, A:E422, A:A423, A:K424, A:R425, A:E426, A:Y427, A:E428, A:E429, A:R430, A:F431, A:N432, A:G433, A:K434, A:K435, A:K436, A:R437, A:E438, A:Y439, A:E440, A:E441, A:R442, A:F443, A:N444, A:G445, A:C446, A:L447, A:K448, A:K449, A:G450, A:L451, A:S452, A:G453, A:G454, A:T455, A:P456, A:S457, A:K458, A:S459, A:A460, A:V461, A:K462, A:K463, A:L464, A:S465, A:G466, A:G467, A:T468, A:P469, A:S470, A:K471, A:S472, A:A473, A:V474, A:E475	57	0.775
4	A:V25, A:K26, A:F28, A:E29, A:E30, A:T31, A:F32, A:V34, A:T35, A:A36, A:A37, A:A38, A:P39, A:V40, A:A41, A:V42, A:A43, A:A44, A:A45, A:G46, A:A47, A:A48, A:P49, A:A50, A:G51, A:A52, A:A53, A:V54, A:E55, A:A56, A:A57, A:E58, A:E59, A:Q60, A:S61, A:E62, A:F63	37	0.701
5	A:D64, A:V65, A:I66, A:L67, A:E68, A:A69, A:A70, A:G71, A:D72, A:K73, A:K74, A:I75, A:K102, A:P103, A:L104, A:L105, A:E106, A:K107, A:V108, A:A109, A:K110, A:E111, A:A112, A:A113, A:D114, A:E115, A:A116, A:K117, A:A118, A:K119, A:L120, A:E121, A:A122, A:A123, A:G124, A:A125, A:T126, A:V127, A:T128, A:V129, A:K130, A:E131, A:A132, A:A133, A:A134, A:K135, A:N136, A:P137, A:R138, A:E139, A:T140, A:G232, A:P233, A:G234, A:P235, A:G236, A:R271, A:G272, A:P273, A:G274, A:P275, A:G276, A:K277, A:H278, A:R279, A:P295, A:G296	67	0.691
6	A:K344, A:V398, A:T400, A:L401, A:Q402, A:V403, A:D404, A:G405, A:K406, A:K407, A:L408, A:M409, A:G410, A:S411, A:S412, A:P413, A:Y414, A:Q415, A:R416, A:H417, A:R418	21	0.624
7	A:L4, A:S5, A:T6, A:D7, A:E8, A:L10	6	0.622
8	A:A371, A:G372, A:P373, A:G374, A:P375, A:G376, A:G377, A:S378, A:A379, A:A380, A:P381, A:A382, A:A383, A:K392	14	0.53

**Table 9:** Docking of vaccines with immune receptors and MHC molecules.

Features	MERV-MHCI	MERV-MHCII	MERV-TLR4
HADDOCK Score	217.3 ± 14.2	179.4 ±27.3	213.6 ± 14.6
Cluster Size	5	3	7
Van der Waals energy	−40.8 ± 3.9	−71.1 ± 2.25	−41.7 ± 1.3
Desolvation energy	−1.41 ± 0.7	−10.7 ± 4.81	−0.57 ± 3.35
Electrostatic energy	−61.8 ± 9.5	−267.1 ± 24.8	−65.1 ± 23.8
RMSD from the overall lowest energy structure	35.6 ± 0.3	10.3 ± 0.5	50.4 ± 0.1
Buried surface area	2211.9 ± 100.2	2871.6 ± 60.1	2171.9 ± 121.9
Z-Score	−1.2	−0.9	−1.7
Restraint violation energy	2246.2 ± 162.9	3724.6 ± 152.4	2920.9 ± 179.4

## Data Availability

Not applicable.
